# Anthelmintic Resistance of Strongyle Nematodes to Ivermectin and Fenbendazole on Cart Horses in Gondar, Northwest Ethiopia

**DOI:** 10.1155/2017/5163968

**Published:** 2017-02-07

**Authors:** Zewdu Seyoum, Alemu Zewdu, Shimelis Dagnachew, Basazinew Bogale

**Affiliations:** Department of Paraclinical Studies, Faculty of Veterinary Medicine, University of Gondar, Gondar, Ethiopia

## Abstract

A study was conducted from November 2015 to April 2016 to determine fenbendazole and ivermectin resistance status of intestinal nematodes of cart horses in Gondar, Northwest Ethiopia. Forty-five strongyle infected animals were used for this study. The animals were randomly allocated into three groups (15 horses per group). Group I was treated with fenbendazole and Group II with ivermectin and Group III was left untreated. Faecal samples were collected from each cart horse before and after treatment. Accordingly, the reduction in the mean fecal egg count at fourteen days of treatment for ivermectin and fenbendazole was 97.25% and 79.4%, respectively. It was significantly different in net egg count between treatment and control groups after treatment. From the study, resistance level was determined for fenbendazole and suspected for ivermectin. In addition, a questionnaire survey was also conducted on 90 selected cart owners to assess their perception on anthelmintics. In the survey, the most available drugs in the study area used by the owners were fenbendazole and ivermectin. Most respondents have no knowledge about drug management techniques. Hence, animal health extension services to create awareness regarding anthelmintic management that plays a key role in reducing the anthelmintic resistance parasites.

## 1. Introduction

Gastrointestinal helminth parasite infection is a major militating factor against profitable animal production in the world over [1]. Horses among most domestic animals have been reported to be more susceptible to a large number of parasites and may harbor different species at a given time [2]. An apparently healthy horse can harbor over half a million gastrointestinal parasites such as protozoa, trematodes, cestodes, and nematodes [3]. This is because the gastrointestinal tract provides a suitable environment for the survival and proliferation of many of these parasites [4].

There are multiple ways to protect horses from many internal parasites that affect them. Every horse owner has different management practices for their herd of horses. Pasture maintenance, anthelmintic usage, and the development of sound herd health plans are just a few ways to maintain the health of the horse in regard to internal parasites. Various parasites, such as large and small strongyles and ascarids, have proven to be problematic in the horse industry [5].

In equine production throughout the world, the use of antiparasitic drugs to control internal and external parasites is a widespread practice. The number of domestically available broad spectrum anthelmintic drugs has increased since 1960s [6]. Several anthelmintics with different modes of action are available in the market for the control of helminthosis [7]. However, the prevalence of anthelmintic-resistant intestinal parasite is a rapidly growing problem in the equine industry [8]. Ivermectin is an analogue of avermectin, which belongs to a family of 16-membered macrocyclic lactones. It is known to increase membrane permeability to chloride ions, possibly as a result of their interaction with chloride ion channels. Its broad spectrum of activity and wide safety margin has made it the drug of choice for nematode and arthropod parasitism in cattle, sheep, goat, swine, and horses [9].

Resistance to anthelmintic medication of horse strongyles, especially of those belonging to the subfamily Cyathostominae, is a worldwide phenomenon [10]. Horse strongyles resistant to benzimidazoles (BZ) and tetrahydropyrimidines (Pyrantel salt, PYR) are well known [11]. The efficacy of macrocyclic lactones (ML) against horse strongyles is high [12]. However, resistance to ML has been described in both horses and donkeys [13].

There are various methods to determine parasite load and identification in the horse industry [14]. Faecal Egg Counts (FEC) and Faecal Egg Reduction Counts (FECR) are considered the simplest and least expensive options to determine the parasite load and how effectively the anthelmintic class being used is reducing the infestation [15]. Several studies have suggested that the faecal egg count reduction test (FECRT) is the gold standard in vivo screening test to detect anthelmintic resistance [16].

The prevalence and impact of strongyle parasites have been studied in many parts of our country [17]. However, the problem of anthelmintic resistance in strongyle horse parasites has not been investigated yet. Assessing the situation in horses, it will be taken into account that parasites are nearly always a subclinical problem, taking into consideration that most Gondar town cart owners follow frequent deworming strategies for treatment and prevention of parasites, and these routine repeated actions open a door for possible resistance.

Therefore, the objectives of this study were

- to investigate the occurrence and the level of anthelmintic resistance of intestinal strongyle nematodes to ivermectin and fenbendazole in Gondar town cart horses,
- to assess the perception of cart horse owners regarding anthelmintic usage and their complaint on the efficacy of commonly used anthelmintics.

## 2. Materials and Methods

### 2.1. Study Area

The study was conducted in Gondar town, Northwest Ethiopia, from November 2015 to March 2016. Gondar town is the capital town of North Gondar Administrative zone, which is located in the Amhara National Regional state. It is found at 740 km away from Addis Ababa to Northwest direction. It is located on 35°7′N and 13°8′E and lies at an altitude of 2,200 meters above sea level. The annual mean minimum and maximum temperature of the area varies between 12–17°C and 22–30°C, respectively [18]. North Gondar zone has an estimated livestock population of cattle 2,771,701; sheep 815,716; goats 1,251,867; horses 27,248; mules 9,695; and donkeys 376,841 and the total number of cart horses in Gondar town is 1,257 according to Gondar town cart horse association (2015).

### 2.2. Study Population and Sampling Technique

The study was conducted on cart horses using simple random sampling technique. One hundred forty cart horses were selected randomly using a lottery system from the cart horse population (1, 257) in the town. Age of the study horses was determined based on owner response and dental eruption [19]. Body condition was recorded as poor and good based on the appearance of the animal and manual palpation of the spinous and transverse process [20]. The study horses were local breeds and mainly kept for human transportation and packing purpose under extensive and intensive management system.

### 2.3. Experimental Design and Methodology

A field experimental study design was conducted to assess the anthelmintic resistance of strongyle nematodes of horses to ivermectin and fenbendazole in Gondar town. For this purpose, faecal specimen was collected from the study animals and subjected for flotation analysis using the McMaster method [21]. Initially, from 140 diagnosed animals 45 naturally strongyle nematode infected animals were identified and allocated for experimental study based on the presence of strongyle eggs in their faeces (EPG ≥ 150 eggs/gram of faeces). Then, horses were grouped into three experimental groups (15 horses per group). Accordingly, Group I horses were treated with fenbendazole, Group II horses treated with ivermectin and Group III: left untreated as positive control. The faecal egg count was determined before (at day 0) and after treatment (at day 14). Thus, the anthelmintic resistance of the tested drugs was determined using a faecal egg count reduction test (FECRT) [22].

### 2.4. Questionnaire Survey

A pretested questionnaire was administered to cart horse owners/managers to assess their perception about the parasite control strategies in horses, anthelmintic usage, and their complaint on the efficacy of commonly used anthelmintics. The questions addressed general management of horse, anthelmintic treatment, dosing frequency, if treatment includes all horses, efficacy of drugs after treatment, type of drugs used, and rotation.

### 2.5. Sample Collection and Sampling Procedures

Faecal samples were collected directly from the rectum and sometimes freshly voided faeces from selected animals using gloves before and after treatment. During sample collection the date, body condition, working type, age, and management system were properly recorded corresponding to the animal identity. The samples were placed in an ice box and transported to University of Gondar, Faculty of Veterinary Medicine, Parasitology Laboratory for faecal examination.

The samples were kept in a refrigerator at +4°C if immediate processing was not possible, but it had been processed within 48 hours. All collected faecal samples were processed using a simple flotation technique with NaCl solution. A qualitative and quantitative faecal examination was made to identify strongyle nematode eggs and to determine the level of infestation, respectively. The faecal egg count (eggs/gram) was considered as a quantitative indicator of infestation level, and it was determined by McMaster technique. Thus, 2-3 gm of faecal matter was mixed in 28 ml of saturated NaCl solution with a lower detection limit of 50 eggs per gram of faeces [23].

### 2.6. Anthelmintic Resistance Test

Cart horses (*n* = 45) aged from 6 years to 12 years and these animals which transport both human and materials were selected to determine the anthelmintic resistance of intestinal strongyle nematodes of horses against ivermectin and fenbendazole drugs. All selected cart horses were with ≥150 eggs per gram of faeces. After doing the faecal egg count (FEC) of strongyle eggs, the horses were randomly allocated into three experimental groups: ivermectin treated group, fenbendazole treated group, and untreated control group (each group contained 15 horses). The dosage and route of application were based on the manufacturers' recommendation as described in Table 1. Faecal samples were collected again 14 days posttreatment from all animals included in the experiment.

### 2.7. Faecal Egg Count Reduction Analysis

The faecal egg count reduction test (FECRT) was used for the evaluation of anthelmintic efficacy/resistance. This method can be adapted for use as a screening agent for veterinarians and producers to identify less than the desired clearance of the parasites after anthelmintic treatment [24]. The resistance of the drugs was tested according to the World Association for the Advancement of Veterinary Parasitology (WAAVP) recommendations for the detection of anthelmintic resistance in horses and ruminants [22] by the percentage reduction of mean egg excretion on the 14-day posttreatment; FECR% = 100(1 − *X*_*t*_/*X*_*c*_), where *X*_*t*_ and *X*_*c*_ are arithmetic means of EPG in the treated (*t*) and control (*c*) groups at day 14 posttreatment. Resistance is present (*R*) if FECR < 90% and the LCL 95% < 90%; resistance is suspected (*S*) if FECR ≥ 90% and/or LCL 95% < 90%; and no resistance (*N*) if FECR ≥ 90% and/or LCL 95% > 90%.

### 2.8. Data Analysis

After faecal analysis, the raw data were recorded with predesigned format and entered into Microsoft excel spreadsheet. Data was analyzed using SPSS software, version 20. Descriptive statistics (means, standard deviation, and reduction percentages) were calculated. Then, ANOVA was used to compare the mean EPG of each experimental group. The effectiveness of the different anthelmintics was evaluated by computing the mean faecal egg count reduction for each treatment group. Computation of the arithmetic mean, percentage of reduction and 95% upper and lower confidence limit, and the findings were interpreted as described by Coles et al. [22]. The result of the questionnaire survey was analyzed by using descriptive statistics to compute or calculate frequency of responses and percentage to summarize the data. Probability (*P*) value less than 0.05 was used to determine the level of significance.

## 3. Results

### 3.1. Questionnaire Survey

The analysis of the respondents showed that chemotherapy was the most important approach (91.1%) towards the control of parasitic diseases. Of the respondents, 62.2% managed their cart horses extensively, whereas 37.8% have not had grazing access to their animals (intensively managed). Similarly, 81.1% and 18.9% of the respondents stated that they have been treating their animals using fenbendazole and ivermectin drugs, respectively. 48.9% and 18.9% of the respondents have also reported that the drugs were bought from private pharmacies and open markets, respectively. The remaining respondents (32.2%) stated that they have found anthelmintics from private and public vet clinics. Moreover, 70% of respondents declared that their animals are treated by themselves, 22% by veterinarians, and 8% by another person. Further, 63.3% of the respondents explained that they did not estimate the weight of the horses to determine the dosage of anthelmintic administered.

Regarding reasons for anthelmintic uses: 56.7% of the respondents stated that they have administered anthelmintics related to general disease symptoms (emaciation, roughed hair coat, reduced body condition, and weakness), 28.9% were due to digestive disturbance (diarrhea, reduced appetite), and 14.4% were due to abdominal problems (rolling and abdominal sound).

### 3.2. Prevalence Determination

A total of 140 cart horses were sampled in the study area. From 140 horses, only 32.14% were demonstrated to harbor strongyle nematode eggs in their faecal matter. At the beginning of animal selection, age, body condition, management system, working type, and treatment status were considered as risk factors. However, the only management system was showing a significant association with strongyle infection rate (*P* = 0.019).

### 3.3. Mean Faecal Egg Counts and Percentage Reduction of Faecal Egg Counts

**Figure 1:**
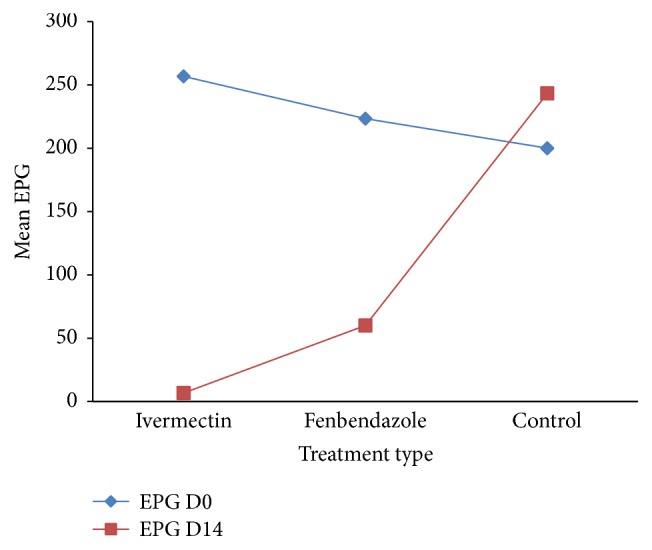
Mean EPG at day 0 and EPG at day 14 with the three experimental groups before and after treatment. EPG D0 = EPG day 0 and EPG D14 = EPG day 14.

The mean pre- and posttreatment faecal egg count and the percentage of faecal egg count reduction and the lower and upper 95% confidence limit for each group of anthelmintic drugs tested were summarized in Table 2. The percentage reduction of faecal egg count for ivermectin and fenbendazole was 97.25% and 79.4%, respectively. Although ivermectin had a fecal egg count reduction percentage greater than 95%, the lower confidence limit of 95% is less than 90%. Based on the result, it can be concluded that ivermectin is suspected for the development of resistance against intestinal strongyle nematode. From the analysis of percentage reduction of faecal egg counts, fenbendazole was found to be under the resistance level of status. The result of the faecal egg count reduction and the lower confidence limit 95% of this drug were less than 90%. From the analysis, ivermectin treated horses recorded significantly (*P* < 0.05) lower mean egg count than fenbendazole treated and untreated control groups of horses. Fenbendazole treated horses also showed significantly (*P* < 0.05) lower egg output than untreated groups after treatment (Figure 1).

## 4. Discussion

The result of the questionnaire survey in this study focused on probing internal parasite control and management practices of horse owners and the parasite resistance problem. It is likely to studies done in Germany [25], UK [26] and Denmark [27]. The majority of (62.2%) the respondents were managing their cart horses extensively and the result indicated that management system has a significant association with strongyle infection. This could be associated with the fact that animals with access to grazing are more exposed to strongyle nematode larvae than indoor animals, which agreed with the study done by Lind et al. [28], Hinney et al. [25], and Nielsen et al. [27]. in European countries.

According to the respondents, in the questionnaire about the use of anthelmintics, the treatment plan was usually designed by themselves (70%), veterinarians (22%), or others (8%) and the weights of horses were evaluated using visual appraisal (56%), by prescription (16%), and by asking another person (28%). Given this possible bias and other responses about the methods used to estimate body weights, it appears that this practice may be a cause of the incorrect dosage of anthelmintics, which may lead to increased parasitic resistance. This is supported by the report of Brady and Nichols [5].

In this attempt, the overall prevalence of strongyle nematode infection in horses was found to be 32.14%. This is in accordance with the finding of Samuel et al. [29] who reported 36.6% infection in studying horses. However, our finding is lower than the work of Alemayehu and Etaferahu [30] who reported 60.8%. The lower prevalence in this study could be due to the fact that study horses were cart horses which have less exposure to the nematode larvae in the field and in some cases horses have been totally restricted from grazing. Moreover, this may be due to the sample size, study type, and season of the year when the study was conducted.

The result showed that the mean FECRT values of fenbendazole and ivermectin were 79.4% and 97.25% with lower 95% confidence interval of 66.5% and 78.59%, respectively. Consequently, the FECR test showed that the anthelmintic resistance was developed and suspected for fenbendazole and ivermectin, respectively. According to Coles et al. [22], the resistance of nematodes in domestic animals can be declared when the percentage reduction in egg counts is less than 95% and/or the lower 95% confidence level is less than 90%. If only one of the two criteria is met, resistance is suspected. There has been no available information about anthelmintic resistance of nematodes in equines in Ethiopia. Most research works regarding anthelmintic resistance and efficacy for GIT nematodes in Ethiopia have been concerned in small ruminants. The resistance of nematode observed in the present study on fenbendazole might be due to the prolonged, frequent, and irrational use and improper dosage. The present finding supports the reports from European countries by Wirtherle et al. [31] and Traversa et al. [32].

In this study, fenbendazole had lower (<95%) faecal egg count reduction percentage and the 95% confidence level is less than 90%, which indicated that strongyle nematodes are resistant to fenbendazole. This may be due to frequent usage, species of the parasite, and poor management system. This report is in agreement with a field study done by Lind et al. [28] in Sweden and Cernea et al. [33] in Romania: the FBZ-treated groups met the criteria for resistance. In contrast to the present study, studies carried out in Romania by Traversa et al. [34] showed the effectiveness of FBZ against horse strongyles (99.49%). They reported that horses have been dewormed once per year or less or never. This may probably be the reason why the resistance against FBZ was not found in these reports.

Ivermectin had a faecal egg count reduction percentage greater than 95% and the lower confidence limit at 95% is less than 90% in our study. Hence, the result indicated that ivermectin is suspected for the development of resistance against intestinal strongyle nematode. This might be due to the quality of the available drug and genetic/strain of the parasite. This finding is in agreement with a number of previous publications from farm horses, in Germany [35] and in USA [12]. They reported that reduction of activity of ivermectin and moxidectin against small strongyles seems to be due to the survival of some of the luminal immature stages in the large intestines after treatment.

In the present study, based on the analysis, ivermectin resistance has been suspected. This is inconsistent with the report of Papadopoulos et al. [36] who explained that faecal egg reduction following ivermectin treatment was reported to be significantly reduced and resistance was not detected in Greece. This was due to the fact that the monitoring activity applied on the efficacy of ivermectin against intestinal strongyles was very high. This difference in reduced efficacy in this study area may be due to drug usage strategies, biology of the parasite, and the quality of the available anthelmintic drugs.

## 5. Conclusion and Recommendations

Both fenbendazole and ivermectin were the common anthelmintic drugs used by Gondar town cart horse owners to control parasitic infestation. However, the current finding indicated that there was a development of fenbendazole resistance, suspecting ivermectin resistance by strongyle nematode of cart horses. Therefore, based on the above conclusions the following recommendations are forwarded.

- Periodic epidemiological studies are essential to accurately establish the nematode infection intensity and prevalence.
- It is important that farmers and veterinarians found a balance between achieving good parasite control and the sustainability of their control strategies to keep the effectiveness of available anthelmintic drugs.
- Creating awareness of the owners about resistance development of these drugs, avoidance of frequent dosing and underdosing, and also alternation with other anthelmintic drugs could be helpful.
- Further studies are needed to determine the anthelmintic resistance status of the different species of GINs in horses in different areas of Ethiopia.

## Figures and Tables

**Table 1:** Details of anthelmintic drugs used in the FECRT for resistance evaluation.

Trade name	Generic name	Manufacturer	Dose per kg body weight	Route of administration
Rangfebenda®	*Fenbendazole*	CIPLA LTD. Pharmaceutical Co., India	7.5 mg/kg	With grain feed
Ivervic®	Ivermectin	Shenyang Sunvictor Pharmaceutical Co. Ltd./China	0.2 mg/kg	Orally

**Table 2:** Pre- and posttreatment fecal egg count, standard deviation, and percent reduction in cart horses.

Sample size	Treatment group	EPG at D0 mean ± SD	EPG at D14 mean ± SD	Reduction in %	LCL	UCL	Remark
15	Ivermectin	260 ± 54.11	6.67 ± 25.82^a^	97.25	78.59	99.65	Suspect
15	Fenbendazole	223 ± 59.36	50 ± 65.46^b^	79.4	66.50	89.96	Resistance
15	Control	200 ± 53.45	243 ± 84.23^c^	NA	NA	NA	

SD: standard deviation, NA: not applicable, D0: day 0, and D014: day 14. ^a, b, c^Statistically different at *P* < 0.05.
